# The Discriminatory Ability of Renalase and Biomarkers of Cardiac Remodeling for the Prediction of Ischemia in Chronic Heart Failure Patients With the Regard to the Ejection Fraction

**DOI:** 10.3389/fcvm.2021.691513

**Published:** 2021-07-29

**Authors:** Dijana Stojanovic, Valentina Mitic, Miodrag Stojanovic, Dejan Petrovic, Aleksandra Ignjatovic, Maja Milojkovic, Olivera Dunjic, Jelena Milenkovic, Vladmila Bojanic, Marina Deljanin Ilic

**Affiliations:** ^1^Institute of Pathophysiology, Faculty of Medicine, University of Nis, Nis, Serbia; ^2^Department of Cardiovascular Rehabilitation, Institute for Treatment and Rehabilitation “Niska Banja”, Niska Banja, Serbia; ^3^Department of Medical Statistics and Informatics, Faculty of Medicine, University of Nis, Nis, Serbia; ^4^Center of Informatics and Biostatistics in Healthcare, Institute for Public Health, Nis, Serbia; ^5^Department of Internal Medicine, Faculty of Medicine, University of Nis, Nis, Serbia

**Keywords:** renalase, discriminatory ability, prediction of ischemia, cardiac remodeling biomarkers, heart failure, HFrEF

## Abstract

**Background:** Renalase has been implicated in chronic heart failure (CHF); however, nothing is known about renalase discriminatory ability and prognostic evaluation. The aims of the study were to assess whether plasma renalase may be validated as a predictor of ischemia in CHF patients stratified to the left ventricular ejection fraction (LVEF) and to determine its discriminatory ability coupled with biomarkers representing a range of heart failure (HF) pathophysiology: brain natriuretic peptide (BNP), soluble suppressor of tumorigenicity (sST2), galectin-3, growth differentiation factor 15 (GDF-15), syndecan-1, and cystatin C.

**Methods:** A total of 77 CHF patients were stratified according to the LVEF and were subjected to exercise stress testing. Receiver operating characteristic curves were constructed, and the areas under curves (AUC) were determined, whereas the calibration was evaluated using the Hosmer-Lemeshow statistic. A DeLong test was performed to compare the AUCs of biomarkers.

**Results:** Independent predictors for ischemia in the total HF cohort were increased plasma concentrations: BNP (*p* = 0.008), renalase (*p* = 0.012), sST2 (*p* = 0.020), galectin-3 (*p* = 0.018), GDF-15 (*p* = 0.034), and syndecan-1 (*p* = 0.024), whereas after adjustments, only BNP (*p* = 0.010) demonstrated predictive power. In patients with LVEF <45% (HFrEF), independent predictors of ischemia were BNP (*p* = 0.001), renalase (*p* < 0.001), sST2 (*p* = 0.004), galectin-3 (*p* = 0.003), GDF-15 (*p* = 0.001), and syndecan-1 (*p* < 0.001). The AUC of BNP (0.837) was statistically higher compared to those of sST2 (DeLong test: *p* = 0.042), syndecan-1 (DeLong: *p* = 0.022), and cystatin C (DeLong: *p* = 0.022). The AUCs of renalase (0.753), galectin-3 (0.726), and GDF-15 (0.735) were similar and were non-inferior compared to BNP, regarding ischemia prediction. In HFrEF patients, the AUC of BNP (0.980) was statistically higher compared to those of renalase (DeLong: *p* < 0.001), sST2 (DeLong: *p* < 0.004), galectin-3 (DeLong: *p* < 0.001), GDF-15 (DeLong: *p* = 0.001), syndecan-1 (DeLong: *p* = 0.009), and cystatin C (DeLong: *p* = 0.001). The AUC of renalase (0.814) was statistically higher compared to those of galectin-3 (DeLong: *p* = 0.014) and GDF-15 (DeLong: *p* = 0.046) and similar to that of sST2. No significant results were obtained in the patients with LVEF >45%.

**Conclusion:** Plasma renalase concentration provided significant discrimination for the prediction of ischemia in patients with CHF and appeared to have similar discriminatory potential to that of BNP. Although further confirmatory studies are warranted, renalase seems to be a relevant biomarker for ischemia prediction, implying its potential contribution to ischemia-risk stratification.

## Introduction

Chronic heart failure (CHF) represents a complex clinical syndrome caused by various etiological factors, leading to structural and/or functional deterioration in the ejection of blood and/or ventricular filling, during stress or at rest (1, 2). Its prevalence depends on the applied study design, but in developed countries, heart failure (HF) accounts for approximately 1 to 2% of adults, increasing to more than 10% in the population older than 70 years (1). Indeed, the outcome of HF has been notably improved, yet its absolute mortality rate remains at 50% within 5 years of diagnosis (2).

For these reasons, there is a remarkable quest on the part of novel biomarkers or multiple biomarker strategies that may prove their diagnostic and/or prognostic benefits in HF (1, 2). According to the 2016 European Society of Cardiology (ESC) guidelines (1), there is still no substantial evidence to fully justify the clinical employment of biomarkers of myocardial remodeling, for example, sST2 and galectin-3. However, the 2013 American College of Cardiology Foundation/American Heart Association (ACCF/AHA) guidelines suggest the determination of sST2 and galectin-3 in the HF population, declaring them as independently predictive of hospitalization and death and additive to natriuretic peptide levels in their prognostic validity (2).

Testing the hypothesis of an enzyme that remarkably contributes to the maintenance of cardiovascular health, a new protein, derived from the kidneys, subsequently called renalase, has been discovered (3). Renalase was evidenced to be a new class of flavin adenine dinucleotide–containing monoamine oxidases (MAOs) (3–5), being weakly associated with MAO-A (3). The additional research confirmed that heart, liver, pancreas, skeletal, reproductive, and neural tissue may also be medically acceptable sources of renalase (3–5). Plasma renalase concentration is most likely up-regulated by circulating catecholamine levels, aiming to metabolize them (3–5) and significantly improving impaired hemodynamic *in vivo* (3). The most recent research has implicated renalase in numerous cardiovascular pathologies: HF (6, 7), coronary artery disease (CAD), hypertension, diabetes mellitus, and aortic stenosis (8–15). Moreover, substantial evidence showed that functional polymorphisms of the renalase gene were associated with cardiac hypertrophy in patients with aortic stenosis (8) and an increased risk of CAD in the general population (9) and in hemodialyzed patients (10), patients with hypertension and associated CAD (11), patients with unstable angina pectoris and concomitant metabolic syndrome (12), and in patients with stable CAD, presenting with cardiac hypertrophy, ventricular dysfunction, and inducible ischemia (13). Moreover, renalase has been suggested as a prognostic biomarker for ischemia in patients with acute coronary microvascular dysfunction (14) and as a predictor for all-cause mortality in chronic kidney disease (15).

Besides decreasing heart rate and contractility, thereby exerting hypotensive properties, renalase has been postulated to function as a cytokine, providing, presumably, anti-ischemic cytoprotection, independently of its catalytic activity (5). Convincing data now exist that renalase exhibits anti-inflammatory and antiapoptotic actions with the intention of cell survival (5, 16–19).

Based on current knowledge, we wanted to assess the following: whether plasma renalase concentration may be validated as a predictor of ischemia during exercise stress testing in patients with CHF stratified to the left ventricular ejection fraction (LVEF) category and to determine its discriminatory ability coupled with biomarkers representing a range of HF pathophysiology: brain natriuretic peptide (BNP), cystatin C, and cardiac remodeling biomarkers, the soluble suppressor of tumorigenicity (sST2), galectin-3, growth differentiation factor 15 (GDF-15), and syndecan-1 for prediction of ischemia in CHF patients with regard to LVEF.

## Patients and Methods

### Study Design and Participants Enrolment

For this cross-sectional, single-center study, CHF patients were selected from the Institute for Treatment and Rehabilitation Niška Banja, Niška Banja, Serbia. The research methodology complied with the Declaration of Helsinki and was reviewed and approved by two institutional ethics committees: the Faculty of Medicine, Niš, University Niš (12-10580-2/3), and the Institute for Treatment and Rehabilitation Niška Banja, Niška Banja (03-4185/1).

Of 120 chronic HF patients who had been admitted to the institute for the purpose of rehabilitation and had initially been randomized for the trial, the eligible participants [77] were those who had complete medical records, met all the criteria for inclusion, and were willing to participate. Briefly, all patients 18 years or older, previously diagnosed with chronic HF who were clinically stable or in the compensated HF status, without any chest pain were classified as a clinical group. The diagnosis of CHF was previously established according to the current guidelines (1) and required the presence of the symptoms and signs of HF, BNP plasma concentration >35 pg/mL, and relevant structural heart changes. The underlying causes for HF included chronic CAD, previous myocardial infarction (with or without ST elevation), valvular diseases, and cardiomyopathy. However, the exclusion criteria were all comorbidities whose pathophysiology might implicate increased concentrations of evaluated biomarkers: chronic kidney disease, liver cirrhosis, diabetes mellitus, systemic or infectious diseases, malignancies, or patients with neuropsychiatric disorders. Consenting patients underwent a complete medical evaluation within 24 h of hospital admission, which included the survey of their complete medical history, blood sampling, clinical examination, and echocardiography, whereas exercise stress tests were performed within 48 h of admission.

A control group (20) comprised healthy community-based volunteers who were age- and gender-matched to the eligible patients. Participants regarded as “controls” were subjected to all procedures and measurements in the same manner as the clinical group.

### Biochemical and Biomarker Measurement

Peripheral blood samples were taken on admission, and all routine biochemical measurements were obtained using Sysmex XS 1,000, Europe GmbH apparatus. Plasma samples were stored at −80°C until biomarker measurement. Therefore, biochemical and biomarker measurements were all quantified from the same sample of plasma.

Biomarker concentration was obtained by quantitative sandwich enzyme-linked immunoassay technique, using the manufacturer's protocol for each of the seven evaluated biomarkers. We determined all standards and samples in duplicate and calculated the average values. Human renalase was determined using the USCN Life Science Inc., China, commercial enzyme-linked immunosorbent assay (ELISA) kit, with a range of detection between 3.12 and 200 ng/mL, whereas the minimum detectable dose of renalase was less than 1.38 ng/mL. The sensitivity of the assay was outlined as the lowest protein value that could be differentiated from zero. It was evaluated by adding 2 standard deviations to the mean optical density of 20 zero-standard replicates, with a concentration calculation.

Plasma concentrations of human sST2, galectin-3, GDF-15, and cystatin C were all determined using Quantikine® (R&D Systems, Inc., Minneapolis, MN, USA) ELISA kits. Human syndecan-1 plasma concentration was determined using Abcam, ab46506 (United Kingdom), and human BNP using Abcam, ab193694 (United Kingdom).

### Echocardiography Measurement

All participants were subjected to two-dimensional echocardiography using a commercially available system (ACUSON–SEQUOIA 256, New York) following the current guidelines (21). The Simpson's biplane method was used for evaluation of the LVEF and left ventricular (LV) volumes, whereas the dimensions of the left ventricle, left atrium, and LV mass were provided by M mode imaging. Diastolic function was estimated by the E/A ratio as the ratio of the early (E) to late (A) ventricular filling velocities. The obtained E/A ratios <1 were regarded as diastolic dysfunction. Relevant structural heart changes evaluated as LV mass index ≥115 g for males and ≥95 g for females or left atrial dilatation ≥40 mm and/or diastolic abnormality (E/A ratio <0.75 or ≥1.5) were mandatory for the diagnosis of chronic HF. Thereafter, according to the gained echocardiographic parameters, the clinical group was divided into two subgroups: patients with verified LVEF ≤ 45% were classified as HF patients with reduced ejection fraction (HFrEF), whereas patients validated as LVEF >45% were classified as the preserved ejection fraction population (HFpEF).

### Exercise Stress Testing

The exercise stress test was performed to evaluate the patient's physical condition, heart rhythm disturbances, and possibly ischemia and for concluding adjustments of their current medication. Therefore, the inclusion criteria for the exercise stress test were complete cardiovascular stability, regardless of the New York Heart Association (NYHA) class or the etiology of HF. Accordingly, the exclusion criteria were hemodynamic instability, cardiac rhythm abnormalities, or uncontrolled hypertension. Exercise stress tests were performed on a treadmill (Treadmill TM2000 *Megatronic*) following the Bruce protocol, meaning that at every 3-min intervals the treadmill speed and slope were gradually increased (22). Patients were continuously monitored for blood pressure, heart rate, and cardiac rhythm abnormalities, as well as for the occurrence of any symptoms (chest pain, shortness of breath, dizziness, or fatigue). The stress test was performed until patients underwent submaximal exercise, achieving four to five estimated metabolic equivalents of exercise that matched 80% of the predicted peak heart rate for their age. The test was terminated in cases when patients requested to stop because of the development of severe symptoms, serious exercise-induced hypertension (>240/11 mm Hg), cardiac rate impairments, ischemic episode development, or any other of the indicators set out in the guidelines (22). An ST-segment response was evaluated for the determination of ischemia, whereas a test was considered positive if horizontal or downsloping ST-segment depression >1 mm (0.1 mV), duration of 0.08 s, occurred in at least 2 consecutive leads.

Stress echocardiography was performed in cases of guideline-directed indications (22) using the Siemens SC2000 and ergo-bicycle (Schiller) with patients adopting a recumbent posture. During the test, patients were supervised using a 12-lead electrocardiogram (ECG) (Schiller AT 10 plus) for an ST-segment response evaluation or cardiac rhythm disturbances assessment. Indications for terminating stress echocardiography test and the interpretation of the results were as aforementioned.

### Statistical Analyses

The data are presented as mean ± standard deviation or as a frequency and percentages. Differences in demographic, clinical, biochemical, and echocardiographic parameters between groups were tested with the χ^2^ test, *t* test and Mann–Whitney *U* test, analysis of variance, and Kruskal–Wallis test. Receiver operating characteristic (ROC) curves were constructed, and the areas under the ROC curves (AUCs) were determined, whereas the calibration was evaluated using the Hosmer-Lemeshow statistic. DeLong test was used to compare the AUCs of evaluated biomarkers (23). Univariate and multivariable logistic regression analysis was applied to determine the independent predictors and predictors after adjustments for age and comorbidities, for prediction of ischemia in HF patients. The odds ratios (ORs), 95% confidence intervals (CIs), and *p* values for individual variables were obtained. Correlations were assessed using the Person analysis. The level of significance was set at *p* < 0.05. Complete case analysis was performed. All statistical analyses were performed using R software, version 3.0.3 (R Foundation for Statistical Computing, Vienna, Austria) (24).

## Results

### Study Participants

Of 120 HF patients who were initially randomized and underwent clinical and biochemical assessment, samples of 77 HF patients were agreed to be most suitable for the final analysis and were primarily classified, according to their performed LVEF as HFrEF (50 patients) and HFpEF (27 patients). Afterward, their baseline data were compared to the control group and presented in Table 1. Among study patients, significant differences were observed concerning the underlying cause of HF as 75.5% of HFrEF had chronic CAD compared to 48.1% HFpEF patients who presented with chronic CAD (*p* = 0.031). Moreover, the HFrEF subgroup comprised significantly more NYHA III/IV classified patients, compared to HFpEF, which was mostly classified of NYHA I/II patients (*p* < 0.001). Hypertension was significantly more prevalent in HFrEF (94%) compared to HFpEF (88.9%) (*p* < 0.001). In contrast, hyperlipidemia was more prevalent in HFpEF (100%) compared to HFrED (82%) (*p* < 0.001). Regarding lipid profile, only plasma triglycerides values were documented to be statistically higher in HFrEF compared to HFpEF (*p* = 0.049), whereas no differences between total cholesterol levels either high-density lipoprotein or low-density lipoprotein fractions were observed among HFrEF and HFpEF, most likely due to the application of strong lipid-lowering therapy. Concerning biochemical analysis, differences were observed in uric acid (*p* < 0.001) and fibrinogen concentration (*p* = 0.019). Even though mean fibrinogen concentration (3.98 ± 0.91) was the highest in HFrEF participants, its values did not increase above the reference value in our laboratory (4 g/L); therefore, we did not consider it pathologically significant. With regard to therapy upon admission, spironolactone was more prevalently used by HFrEF patients (84%), compared to HFpEF (25.9%), *p* < 0.001, with no significant differences in any other type of therapy. Regarding echocardiographic parameters, presented in Table 1, significant differences were obtained in LV mass index (*p* = 0.001), end-systolic diameter (*p* < 0.001), end-diastolic diameter (*p* < 0.001), interventricular septum diameter (*p* = 0.001), posterior wall diameter (*p* = 0.001) and diastolic dysfunction E/A (*p* = 0.001).

**Table 1:** Baseline characteristics of study groups.

**Parameter**	**HFrEF (≤45%)**	**HFpEF (>45%)**	**Control group**	***p***
Mean age in years	60.74 ± 10.28	63.63 ± 9.02	59.40 ± 10.95	0.379^1^
Male, %	77.8	74.0	70.0	0.283^3^
**Heart failure cause %** ^†^				
Coronary artery disease	75.5	48.1		**0.031** ^3^
Myocardial infarction	59.3	35.3		0.784^3^
Valvular heart disease	36.0	37.0		>0.999^3^
Cardiomyopathy	71.4	70.4		>0.999^3^
**Hemodynamic**, mm/Hg				
Systolic blood pressure	126.80 ± 14.20	128.89 ± 22.16	119.00 ± 6.99	0.275^1^
Diastolic blood pressure	78.50 ± 9.10	78.52 ± 8.06	77.00 ± 4.83	0.868^1^
**NYHA functional class** ^†^				
I	18.0	81.5		**<0.001^3^**
II	44.0	18.5		
III	22.0	0.0		
IV	16.0	0.0		
Family history, %	58.0	70.4	50.0	0.424^3^
Hypertension, %	94.0	88.9	0.0	**<0.001^4^**
Hyperlipidemia, %	82.0	100.0	20.0	**<0.001^4^**
Obesity, %	62.0	63.0	30.0	0.150^3^
Smoking history, %	46.0	51.9	30.0	0.487^3^
**Laboratory parameters**				
Total cholesterol, mmol/L	4.88 ± 1.30	4.61 ± 1.50	5.26 ± 1.25	0.253^2^
LDL, mmol/L	3.04 ± 1.14	2.95 ± 1.19	3.43 ± 1.03	0.346^2^
HDL, mmol/L	1.03 ± 0.24	1.03 ± 0.23	1.18 ± 0.30	0.231^2^
Triglycerides, mmol/L	1.79 ± 0.70^a^	1.50 ± 0.76	1.45 ± 0.74	**0.049^2^**
BUN, mmol/L	8.38 ± 5.89	6.24 ± 1.63	5.64 ± 1.97	0.067^2^
Creatinine, μmol/L	120.22 ± 47.25	104.56 ± 22.31	73.81 ± 6.15	0.058^2^
eGFR, mL/min/1.73m^2^	59.64 ± 15.64	64.42 ± 15.40	65.37 ± 13.36	0.391^2^
Uric acid, mmol/L	436.07 ± 121.51^a,b^	323.53 ± 89.43	332.36 ± 102.76	**<0.001^2^**
Fibrinogen, g/L	3.88 ± 0.91^b^	3.71 ± 0.61^b^	3.13 ± 0.52	**0.019^2^**
C-reactive protein, mg/L	1.92 ± 5.61	0.44 ± 2.31	2.40 ± 5.06	0.306^2^
**Therapy upon admission, %^†^**				
ACEI/ARB	92.0	81.5		0.318^3^
Amiodarone	44.0	22.22		0.099^3^
Beta blocker	96.0	96.3		>0.999^3^
Calcium channel blocker	18.0	18.0		0.217^3^
Diuretic	84.0	66.47		0.144^3^
Spironolactone	84.0	25.9		**<0.001^3^**
Statin	98.0	96.3		>0.999^3^
**Echocardiographic measurement**				
LVMI (g/m^2^)	155.56 ± 35.12^a,b^	116.67 ± 25.89	82.1 ± 8.98	**0.001** ^2^
ESD (mm)	49.34 ± 9.89^a,b^	36.6 ± 3.28	30.98 ± 2.76	**<0.001** ^2^
EDD (mm)	64.56 ± 5.98^a,b^	53.4 ± 5.09	48.87 ± 2.45	**<0.001** ^2^
IV septum (mm)	12.87 ± 1.5^a,b^	11.08 ± 1.44	10.5 ± 1.31	**0.001** ^2^
Posterior wall (mm)	9.15 ± 1.77^a,b^	10.06 ± 1.08	9.25 ± 0.87	**0.001** ^2^
E/A	0.87 ± 0.22^a,b^	0.77 ± 0.21	1.1 ± 0.2	**0.001** ^2^
**Biomarkers**				
BNP, pg/mL	219.38 ± 159.92^a,b^	94.0 8± 21.42^b^	14.86 ± 7.22	**<0.001** ^2^
Renalase, ng/mL	147.52 ± 29.39^a,b^	122.63 ± 38.61^b^	24.49 ± 4.74	**<0.001** ^2^
sST2, ng/mL	33.42 ± 10.16^a,b^	26.14 ± 7.79^b^	16.06 ± 3.78	**<0.001** ^2^
Galectin-3, ng/mL	28.22 ± 5.12^a,b^	22.48 ± 4.86^b^	17.11 ± 1.29	**<0.001** ^2^
GDF-15, ng/mL	1900.14 ± 571.13^a,b^	1488.99 ± 413.83^b^	542.69 ± 48.22	**0.001** ^2^
Syndecan-1, ng/mL	73.14 ± 11.86^a,b^	56.92 ± 16.54^b^	13.01 ± 3.80	**<0.001** ^2^
Cystatin C, mg/L	1.34 ± 0.41^a,b^	1.14 ± 0.21^b^	0.92 ± 0.05	**0.001** ^2^

*Continous variables are expressed as mean ± standard deviation*,

1 *ANOVA*,

2 *Kruskal-Wallis test*,

3 *Hi-squared test*;

4 *Fisher's exact test; bold values are p < 0.05*,

a *p < 0.05 vs. HFpEF*,

b *p < 0.05 vs. control group*,

† *without control group*.

*HFrEF, heart failure with reduced ejection fraction; HFpEF, heart failure with preserved ejection fraction; NYHA, New York Heart Association; LDL, low density lipoprotein; HDL, high density lipoprotein; BUN, blood urea nitrogen; eGFR, estimated glomerular filtration rate; ACEI, angiotensin-converting enzyme inhibitor; ARB, angiotensin II receptor blocker; LVMI, left ventricular mass index; ESD, end-systolic dimension; EDD, end-diastolic dimension; IV, interventricular septum; BNP, brain natriuretic peptide; sST2, soluble source of tumorigenicity 2; GDF-15, growth differentiation factor 15*.

The mean plasma concentrations of all evaluated biomarkers in study participants are summarized in the same table. Significant differences were evidenced between both subgroups (HFrEF vs. HFpEF) and the control group, for plasma concentrations of BNP (*p* < 0.001), renalase (*p* < 0.001), sST2 (*p* < 0.001), galectin-3 (*p* < 0.001), GDF-15 (*p* = 0.001), syndecan-1 (*p* < 0.001), and cystatin C (*p* = 0.001), respectively. Moreover, a meaningful pattern was recognized, with all concentrations being the highest in HFrEF patients. After these initial findings, further analysis of biomarker plasma concentration, concerning underlying HF etiology (chronic CAD vs. other causes), for total CHF population, as well as for both (HFrEF and HFpEF) subtypes, was performed. However, it did not indicate any significant differences; therefore, we did not include it in the final results.

### Correlation of Renalase With Biomarkers

Table 2 summarizes correlation coefficients between plasma concentrations of renalase and evaluated biomarkers stratified by LVEF category. In HFrEF phenotype, we noted significant positive correlations between plasma renalase and all evaluated biomarker concentrations, as follows: BNP (*p* = 0.004), sST2 (*p* < 0.001), galectin-3 (*p* < 0.001), syndecan-1 (*p* < 0.001), GDF-15 (*p* < 0.001), and cystatin C (*p* < 0.001). Similarly, in HFpEF phenotype, the positive correlations of renalase were obtained relating to all biomarkers of cardiac remodeling: sST2 (*p* < 0.001), galectin-3 (*p* < 0.001), syndecan-1 (*p* < 0.001), GDF-15 (*p* < 0.001), and cystatin C (*p* < 0.001). However, no significant correlations between plasma concentrations of renalase and BNP were obtained in the HFpEF phenotype, as shown in Table 2.

**Table 2:** Correlation coefficients between renalase and biomarkers with regard to the ejection fraction.

**Biomarkers/HF phenotype**		**HFrEF (EF ≤45%)**						**HFpEF (EF >45%)**					
		Renalase	sST2	Gal-3	Syn-1	GDF-15	Cystatin C	Renalase	sST2	Gal-3	Syn-1	GDF-15	Cystatin C
BNP	r	0.343^*^	0.385^**^	0.427^**^	0.337^*^	0.388^**^	0.043	0.344	0.344	0.305	0.521^**^	0.384	0.241
	p	**0.014**	**0.005**	**0.002**	**0.016**	**0.005**	0.763	0.085	0.086	0.130	**0.006**	0.053	0.236
Renalase	r	1	0.891^**^	0.843^**^	0.740^**^	0.860^**^	0.822^**^	1	0.868^**^	0.864^**^	0.922^**^	0.867^**^	0.805^**^
	p		**<0.001**	**<0.001**	**<0.001**	**<0.001**	**<0.001**		**<0.001**	**<0.001**	**<0.001**	**<0.001**	**<0.001**
sST2	r		1	0.907^**^	0.864^**^	0.872^**^	0.678^**^		1	0.813^**^	0.848^**^	0.773^**^	0.790^**^
	p			**<0.001**	**<0.001**	**<0.001**	**<0.001**			**<0.001**	**<0.001**	**<0.001**	**<0.001**
Galectin-3	r			1	0.878^**^	0.823^**^	0.665^**^			1	0.841^**^	0.663^**^	0.701^**^
	p				**<0.001**	**<0.001**	**<0.001**				**<0.001**	**<0.001**	**<0.001**
Syndecan-1	r				1	0.737^**^	0.536^**^				1	0.860^**^	0.759^**^
	p					**<0.001**	**<0.001**					**<0.001**	**<0.001**
GDF-15	r					1	0.760^**^					1	0.763^**^
	p						**<0.001**						**<0.001**

*r-correlation coefficient; bold values are p < 0.05*.

*HFrEF, heart failure with reduced ejection fraction; HFpEF, heart failure with preserved ejection; BNP, brain natriuretic peptide; sST2, soluble source of tumorigenicity 2; GDF-15, growth differentiation factor 15; Gal-3, galectin 3; Syn-1, syndecan-1*.

* *p < 0.05*,

** *p < 0.01*.

### Prognostic Evaluation of Renalase

Table 3 presents the results of testing renalase and evaluated biomarkers in a logistic regression model as predictors for the development of ischemia during exercise stress tests. It was, therefore, confirmed that significant and independent predictors of ischemia in the total HF cohort were shown to be the increased plasma concentrations as follows: BNP (OR = 0.99, 95% CI = 0.982–0.997, *p* = 0.008), renalase (OR = 0.86, 95% CI = 0.761–0.966, *p* = 0.012), sST2 (OR = 0.95, 95% CI = 0.919–0.993, *p* = 0.020), galectin-3 (OR = 0.93, 95% CI = 0.881–0.988, *p* = 0.018), GDF-15 (OR = 0.99, 95% CI = 0.998–1.000, *p* = 0.034), and syndecan-1 (OR = 0.93, 95% CI = 0.889–0.992, *p* = 0.024). Multivariable adjustments, for age and comorbidities, however, revealed that only BNP (OR = 0.99, 95% CI = 0.977–0.997, *p* = 0.010) remained a predictor of ischemia in the total chronic HF clinical group. Similar results are also presented in Table 3, whereas we analyzed risk factors for the prediction of ischemia according to LVEF rate. Significant results were confirmed for HFrEF patients and accordingly are presented in Table 3. Biomarkers whose increased plasma concentration was evidenced as significant and an independent risk factor for prediction of ischemia in HFrEF patients were as follows: BNP (OR = 1.14, 95% CI = 1.057–1.235, *p* = 0.001), renalase (OR = 1.32; 95% 1.152–1.517, *p* < 0.001), sST2 (OR = 1.19, 95% CI = 1.057–1.341, *p* = 0.004), galectin-3 (OR = 1.06, 95% CI = 1.021–1.103, *p* = 0.003), GDF-15 (OR = 1.00, 95% CI = 1.001–1.004, *p* = 0.001), syndecan-1 (OR = 1.09, 95% CI = 1.046–1.136, *p* < 0.001), and presence of chronic CAD (OR = 3.69, 95% CI = 1.349–10.121, *p* = 0.011). Correspondingly, the multivariable regression model, adjusted for the same variables, revealed that only a BNP plasma concentration (OR = 1.16, 95% CI = 1.058–1.278, *p* = 0.002) and chronic CAD (OR = 23.42, 95% CI = 1.028–533.547, *p* = 0.048) represented risk factors for ischemia in the HFrEF subgroup. However, no significant risk factors for the development of ischemia were confirmed in HFpEF; therefore, those results are not presented in the table.

**Table 3:** Univariate and multivariable regression analyses of renalase, BNP, cystatin C and biomarkers of myocardial remodeling for prediction of ischemia in the chronic HF patients.

**Parameters**	**Univariate regression analysis**			**Multivariable regression analysis**		
	**OR**	**95%CI**	***p***	**OR**	**95%CI**	***p***
**Total cohort of chronic HF**						
Age	0.97	0.916–1.039	0.446	0.97	0.984–1.008	0.526
Gender	0.60	0.158–2.273	0.452	1.02	0.993–1.050	0.134
BMI	0.99	0.854–1.156	0.932	1.11	0.394–3.135	0.842
Chronic CAD	1.12	0.295–4.230	0.871	1.08	0.420–2.796	0.869
BNP	0.99	0.982–0.997	**0.008**	0.99	0.977–0.997	**0.010**
Renalase	0.86	0.761–0.966	**0.012**	1.03	0.824–1.278	0.816
sST2	0.95	0.919–0.993	**0.020**	1.01	0.948–1.075	0.767
Galectin-3	0.93	0.881–0.988	**0.018**	1.09	0.715–1.679	0.676
GDF-15	0.99	0.998–1.000	**0.034**	1.00	0.997–1.003	0.822
Syndecan-1	0.93	0.889–0.992	**0.024**	0.95	0.828–1.089	0.457
Cystatin C	0.47	0.121–1.839	0.279	0.98	0.949–1.021	0.400
Hosmer-Lemeshow test *p* = 0.192						
**HFrEF phenotype**						
Age	0.97	0.922–1.018	0.212	1.04	0.970–1.123	0.250
Gender	1.14	0.376–3.455	0.816	0.35	0.041–2.973	0.334
BMI	1.03	0.917–1.165	0.588	0.95	0.791–1.142	0.588
Chronic CAD	3.69	1.349–10.121	**0.011**	23.42	1.028–533.547	**0.048**
BNP	1.14	1.057–1.235	**0.001**	1.16	1.058–1.278	**0.002**
Renalase	1.32	1.152–1.517	**<0.001**	0.98	0.959–1.002	0.069
sST2	1.19	1.057–1.341	**0.004**	0.98	0.947–1.010	0.978
Galectin-3	1.06	1.021–1.103	**0.003**	0.95	0.832–1.078	0.408
GDF-15	1.00	1.001–1.004	**0.001**	1.00	0.998–1.001	0.610
Syndecan-1	1.09	1.046–1.136	**<0.001**	1.01	0.962–1.054	0.777
Cystatin C	0.97	0.947–1.010	0.978	1.00	0.984–1.022	0.789
Hosmer-Lemeshow test *p* = 0.833						

*Multivariable model adjusted for age and comorbidities; bold values are p < 0.05*.

*HF, heart failure; CI, confidence interval; BMI, body mass index; CAD, coronary artery disease; BNP, brain natriuretic peptide; sST2, soluble source of tumorigenicity 2; GDF-15, growth differentiation factor 15*.

### The Discriminatory Ability of Renalase

**Figure 1:**
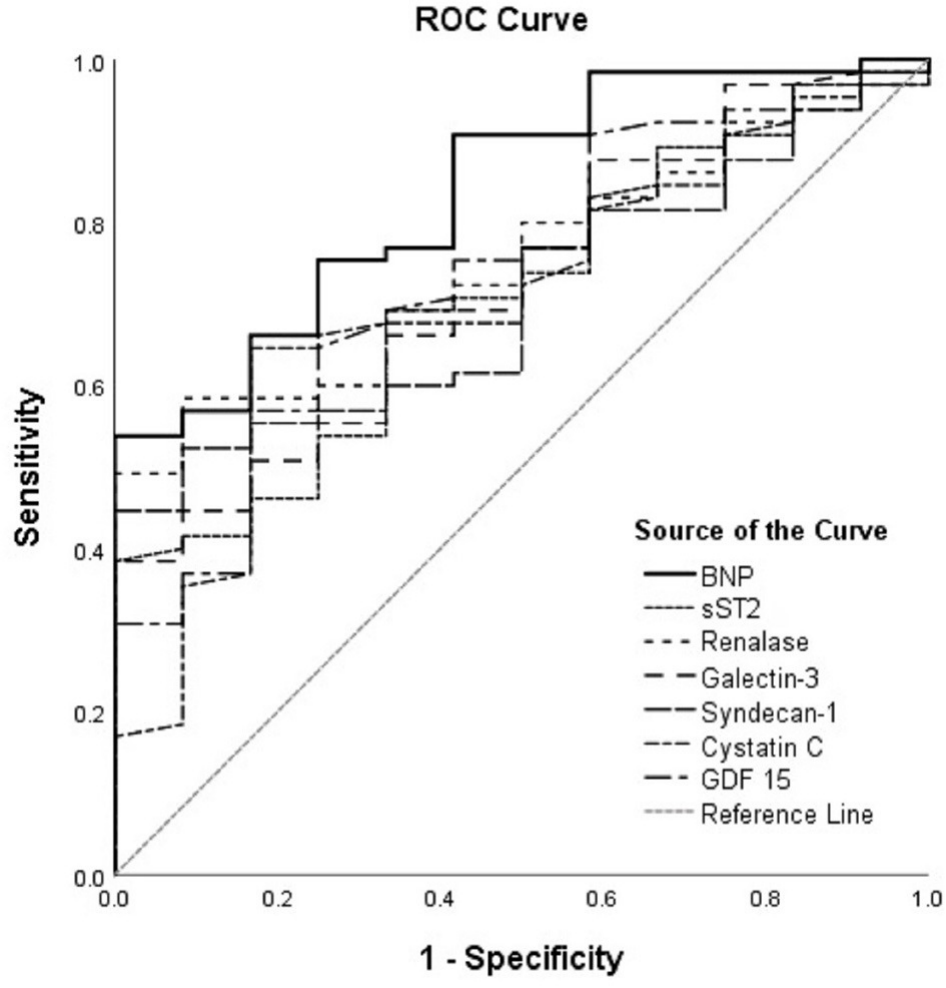
ROC curves of renalase and evaluated biomarkers for the prediction of ischemia in the total heart failure group. The curves should be interpreted with regard to Table 4. AUCs: BNP (0.837), sST2 (0.712), renalase (0.753), galectin-3 (0.726), syndecan-1 (0.709), cystatin C (0.704), GDF-15 (0.735). ROC, receiver operating characteristic; AUC, area under the curve; BNP, brain natriuretic peptide; sST2, soluble source of tumorigenicity 2; GDF-15, growth differentiation factor 15.

**Figure 2:**
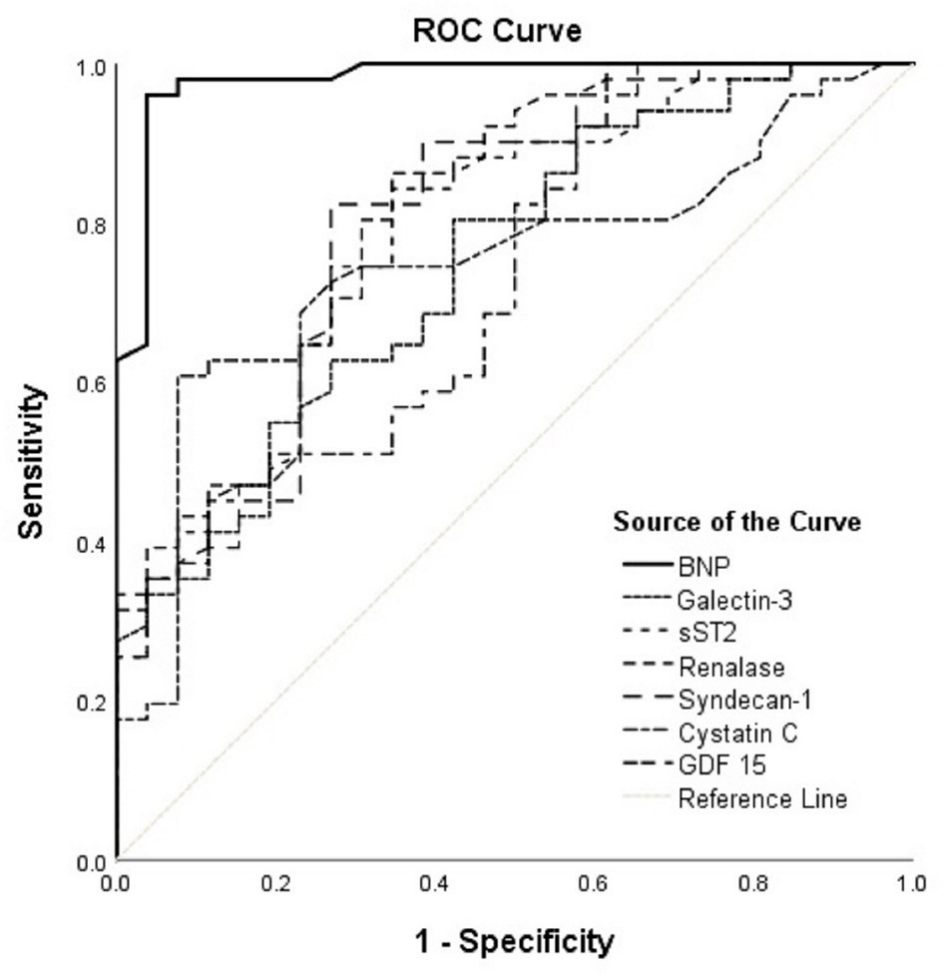
ROC curves of renalase and evaluated biomarkers for the prediction of ischemia in the HFrEF phenotype. The curves should be interpreted with regard to Table 5. AUCs: BNP (0.980), galectin-3 (0.747), sST2 (0.788), renalase (0.814), syndecan-1 (0.801), cystatin C (0.749), GDF-15 (0.731). ROC, receiver operating characteristic; AUC, area under the curve; BNP, brain natriuretic peptide; sST2, soluble source of tumorigenicity 2; GDF-15, growth differentiation factor 15.

The ROC curves of renalase and cardiac remodeling biomarkers for prediction of ischemia during exercise stress testing are shown in Figure 1, for the total cohort of HF patients, and Figure 2, for HFrEF patients. The analysis of discriminatory abilities of evaluated biomarkers for prediction of ischemia should be interpreted with regard to Tables 4, 5. Plasma BNP evidenced the best discriminatory ability for the prediction of ischemia compared to all evaluated biomarkers and demonstrated statistically higher AUC [0.837 (95% CI = 0.729–0.946, *p* < 0.001)] compared to those of the following biomarkers: sST2 (DeLong test: *p* = 0.042), syndecan-1 (DeLong test: *p* = 0.022), and cystatin C (DeLong test: *p* = 0.022). The AUC of renalase [0.753 (95% CI = 0.635–0.871, *p* = 0.006)] was lower compared to that of BNP, but not statistically significant, and was significantly higher compared to syndecan-1 (DeLong test: *p* = 0.025). Moreover, there were no statistically significant differences in the AUCs of renalase, sST2, galectin-3, GDF-15, and cystatin C. The AUCs of the other biomarkers were as follows: sST2 [0.712 (95% CI = 0.573–0.85, *p* = 0.020)], galectin-3 [0.726 (95% CI = 0.588–0.864, *p* = 0.013)], GDF-15 [0.735 (95% CI = 0.594–0.875, *p* = 0.010)], syndecan-1 [0.709 (95% CI = 0.582–0.836, *p* = 0.022)], and cystatin C [0.704 (95% CI = 0.556–0.853, *p* < 0.001)]. The aforesaid results refer to the total chronic HF study group and are presented in Table 4, Figure 1.

**Table 4:** Areas under the ROC curve for prediction of ischemia in total heart failure group.

**Biomarkers**	**AUC**	**95%CI**	**Standard error**	***p***
BNP	0.837	0.729–0.946	0.055	**<0.001**
Renalase	0.753	0.635–0.871	0.060	**0.006**
sST2	0.712^a^	0.573–0.85	0.071	**0.020**
Galectin-3	0.726	0.588–0.864	0.070	**0.013**
GDF-15	0.735	0.594–0.875	0.072	**0.010**
Syndecan-1	0.709 <^a,b^	0.582–0.836	0.065	**0.022**
Cystatin C	0.704^a^	0.556–0.853	0.076	**<0.001**

*p < 0.05*.

*AUC, Area under the curve; ROC, receiver operating characteristic; CI, confidence interval; SE, standard error; BNP, brain natriuretic peptide; sST2, soluble source of tumorigenicity 2; GDF-15, growth differentiation factor 15. DeLong test was used for comparisons of AUCs*:

a *p < 0.05 vs BNP*,

b *p < 0.05 vs renalase*.

**Table 5:** Areas under the ROC curves for prediction of ischemia in the HFrEF phenotype.

**Biomarkers**	**AUC**	**95%CI**	**Standard error**	***p***
BNP	0.980	0.951–1.000	0.015	**<0.001**
Renalase	0.814^a^	0.712–0.916	0.052	**<0.001**
sST2	0.788^a,c^	0.681–0.895	0.054	**<0.001**
Galectin-3	0.747^a,b^	0.635–0.860	0.058	**<0.001**
GDF-15	0.731^a,b^	0.614–0.848	0.060	**0.001**
Syndecan-1	0.801^a,c^	0.693–0.909	0.055	**<0.001**
Cystatin C	0.749^a^	0.636–0.861	0.057	**<0.001**

*p < 0.05*.

*AUC, Area under the curve; ROC, receiver operating characteristic; CI, confidence interval; BNP, brain natriuretic peptide, sST2, soluble source of tumorigenicity 2; GDF-15, growth differentiation factor 15*.

*DeLong test was used for comparisons of AUCs*:

a *p < 0.05 vs BNP*,

b *p < 0.05 vs renalase*,

c *p < 0.05 vs galectin-3*.

Accordingly, Figure 2 interpretation should be performed with regard to Table 5 and shows results obtained in the HFrEF phenotype. Plasma BNP kept the best discriminatory ability compared to all assessed biomarkers in the HFrEF phenotype and demonstrated statistically higher AUC [0.980 (95% CI = 0.951–1.000, *p* < 0.001)] on the top of the AUCs of other biomarkers, as follows: renalase [0.814 (95% CI = 0.712–0.916; DeLong test: *p* < 0.001)], sST2 [0.788 (95% CI = 0.681–0.895; DeLong test: *p* < 0.004)], galectin-3 [0.747 (95% CI = 0.635–0.860; DeLong test: *p* < 0.001)], GDF-15 [0.731 (95% CI = 0.614–0.848; DeLong test: *p* = 0.001)], syndecan-1 [0.801 (95% CI = 0.693–0.909; DeLong test: *p* = 0.009)], and cystatin C [0.749 (95% CI = 0.636–0.861; DeLong test: *p* = 0.001)]. The discriminatory ability of renalase for ischemia prediction was statistically higher compared to those of galectin-3 (DeLong test: *p* = 0.014) and GDF-15 (DeLong test: *p* = 0.046) and similar to that of sST2. Also, AUCs of sST2 (DeLong test: *p* = 0.026) and of syndecan-1 (DeLong test: *p* = 0.038) were significantly higher compared to that of galectin-3. No statistical significance for observed biomarkers was evidenced in the HFpEF population; therefore, it was not presented in our Tables.

## Discussion

Even though it was first suggested that renalase originates from the kidneys to the extent that it metabolizes catecholamines, lowering blood pressure, heart rate, and contractility, the mechanisms of renalase in the cardiovascular pathophysiology are presumably more complex. The evidence that renalase exhibits marked cytokine properties, protecting cells from ischemic injury and modulating inflammation and apoptosis (5), leads to the presumption of its therapeutic benefits, encouraging further open-ended investigations.

The current study represents an ongoing analysis of the potential role of renalase in CHF patients with regard to the LVEF. Our previous research evidenced that plasma renalase might be a biomarker that would be able to differentiate HFrEF patients from those with midrange and preserved LVEF, concomitantly being strongly associated with increased LV mass index (6). In addition, we confirmed that elevated plasma renalase concentration, when present in chronic HF patients, regardless of the LVEF rate, represented a significant prognostic factor for an increase of biomarkers of cardiac remodeling plasma concentration (7). According to our latest results, renalase may be a valuable prognostic factor for ischemia during exercise stress tests in chronic HF patients, including the patients with LVEF of <45%. Surprisingly, albeit BNP evidenced the best discriminatory potential for ischemia prediction on top of renalase and other evaluated biomarkers in the total HF cohort, it was not statistically significant. Accordingly, renalase, in line with sST2, galectin-3, and GDF-15, clearly demonstrated non-inferiority for ischemia prediction compared to BNP, implying relevance in addition to established risk factors. In the HFrEF phenotype, however, BNP indicated significantly better discrimination for ischemia prediction compared to all evaluated biomarkers, whereas renalase discriminatory potential was similar to that of sST2, but better compared to those of galectin-3 and GDF-15. These results, indeed, provide the scientific rationale for renalase determination in HF patients, ensuring its further inclusion in the comparative biomarker analysis. This is, truly, the very first study to review and confirm the prognostic potential of renalase for ischemia, regarding the ejection fraction stratification. Likewise, impressive evidence has recently implicated renalase as a possible biomarker for ischemia (5, 14, 16–18, 25–27). The obtained findings may add considerably to the growing body of literature in this field.

The most plausible hypothesis of renalase antihypoxic and anti*-*ischemic properties suggests that the renalase secretion of cardiomyocytes is presumably induced by hypoxia and that this response is achieved through activation of the hypoxia-inducible factor 1α (HIF-1α) gene (25). More precisely, it was evidenced that renalase represents a myocardial hypoxia-responsive gene that correlates with HIF-1α expression. The same research indicated that HIF-1α may bind to the promoter of renalase, in order to facilitate its transactivation, promoting cardiac protection against hypoxia (25). The peak of renalase myocardial expression and its serum activity was observed 12 h after ischemia initiation and declined thereafter. The most relevant findings were that the myocardial ischemic lesion area was remarkably enlarged, and the ejection fraction rate significantly decreased in the setting where myocardial renalase expression knockdown preceded the ischemic insult. Indeed, the application of recombinant renalase mitigated the deterioration of cardiac function and structure (25). Accordingly, another study confirmed that, during and after ischemic episodes, diminished myocardial expression of renalase led to aggravation of cardiac failure, confirmed through cardiomyocyte necrosis and apoptosis (16). The important role of renalase in the local heart tissue, as well as its possible roles in different organs, was concluded, proposing renalase as a relevant therapeutic target for ischemic damage (16). More recent research (14) evidenced that renalase was significantly increased in patients with acute coronary microvascular dysfunction presenting with ischemic chest pain, suggesting that renalase elevation was transitory, pointing to a physiological response to ischemia. Nevertheless, the authors nominated renalase as an anti-inflammatory marker and suggested its advantage as a possible biomarker for ischemia (14). Similarly, in the experimental model of ischemia-induced HF, it was evidenced that renalase levels peak in the first week after the ischemic injury, with a subsequent decrease during the follow-up, suggesting that cardiac decompensation seemingly results in subbasal renalase concentration (27). Once again, recombinant renalase administration was proven to lessen ischemic cardiac injury and to hinder a severe fall in LVEF (18), a hypothesis that may be applied to the HFrEF patients in our model.

The same theory has been further confirmed in the experimental model of ischemic kidney injury (17, 26). The conclusion was supported that renalase exerts renal protection in the setting of ischemic acute kidney injury by diminishing inflammation, necrosis, and apoptosis, suggesting the use of renalase as a novel biomarker of ischemic kidney injury (17). Correspondingly, the other study (26) provided evidence that ischemic injury significantly increased renalase kidney cortex expression, *in vitro* and *in vivo*, further concluding that HIF-1α directly up-regulates renalase expression. The authors, however, extended the period of renalase action, beyond its prompt activation, underpinning a delayed ischemic environment. Renalase expression peaked 24 h after the initial ischemic injury, suggesting that renalase presumably has a significant role in the protective mechanisms of delayed and possible chronic ischemia. Moreover, the authors in both studies confirmed the beneficial and protective effects of recombinant renalase therapy.

We have confirmed that HFrEF patients, compared to those with normal or near-normal LVEF, presented with the highest renalase levels within the total HF population and with a multifold increase compared to the controls. This elevation, presumably, represents a physiological reaction to chronic ischemia (hypoxia), intending to diminish oxidative injury and alleviating cardiac remodeling, as seen in experimental models (16–19). In addition, renalase levels presumably rise with the aim of counteracting cardiac remodeling biomarkers cascade, as our results clearly demonstrate in both HF phenotypes. Moreover, it is known that vasoactive peptides, such as BNP, downregulate the sympathetic nervous system in HFrEF, intending to decrease catecholamines production. It may be presumed that renalase and BNP share similar mechanisms of action in catecholamine surge overthrow, resulting in their strong and positive correlation in particular HFrEF phenotype. However, we did not find any significant differences in renalase plasma levels with regard to the etiology of HF, for example, between patients with underlying CAD (ischemic origin) and patients who presented with another etiology (valvular disease or cardiomyopathy) within the unique HFrEF cohort. These findings may be attributed to the fact that the HFrEF subgroup comprised the substantially greater population with underlying chronic CAD (>75%). This may lead to the question as to whether increased renalase levels may be associated with a risk for CAD. Nevertheless, such a link has already been confirmed, considering that genetic testing of renalase rs2576178 polymorphism proved its association with increased risk of CAD development (9). There are more than a few pertinent explanations for renalase elevation in the setting of CHF, particularly HF with reduced LVEF.

Pathophysiologically speaking, HFrEF may be discussed as the site of a hypoxic inflammation, as low LVEF results in poor perfusion and diminished tissue oxygenation. Coupled with that, HIF-1α activation presumably leads to increased renalase synthesis and secretion. Similarly, hypoxia is described as an activator of nuclear factor κβ (NF-κβ), resulting in the inflammatory and apoptotic-gene expression, likely followed by renalase elevation (28). However, it is reasonable to postulate that those transcription factors interact gradually in order to restore or compensate low tissue oxygenation (29), mutually regulating renalase activation. Besides HIF-1α (25, 26) and NF-κβ (5, 28), crucial transcription factors for renalase gene expression are evidenced to be specificity protein 1 (Sp1), signal transducer and activator of transcription 3 (STAT3) and zinc-binding protein 89 (ZBP89) (30).

Substantial evidence revealed that antihypoxic and anti-ischemic features of plasma renalase are achieved by triggering receptor-mediated signal transduction mechanisms such as STAT3, mitogen-activated protein kinase (MAPK), and protein kinase B (AKT), whereas the plasma membrane Ca^2+^-ATPase (PMCA4b) was identified as the receptor for extracellular renalase, also representing a part of the signaling complex (5). In addition to cardioprotection, renalase was validated to inhibit the profibrotic gene expression and phosphorylation of the extracellular signal–regulated kinase 1/2 pathway, therefore preventing adverse cardiac remodeling (5). Furthermore, a hypoxic environment moves the mitochondrial oxidative metabolism toward glucose uptake, resulting in increased glycolysis; therefore, renalase may be secreted in the process of preserving the primary metabolism (8). Coupled with this, higher levels of renalase were previously confirmed in unstable angina pectoris patients, presuming that renalase rises in such conditions, owing to the body's metabolic changes, postponing its elevation grants mitigation of emergency cardiovascular conditions, including CAD (12).

The most recent findings, favoring renalase antihypoxic and anti-ischemic protection, beyond the scope of cardiology, refer to hepatic ischemic injury (31, 32). *In vitro* and *in vivo* confirmed that renalase levels were appropriately responsive to the ischemic liver injury and, more importantly, that renalase serum levels were able to sensitively mirror the severity of an ischemic lesion in the liver (31). The authors also demonstrated that variations in renalase concentration reflected the effects of applied antioxidative therapy, suggesting renalase as a potential biomarker for the complete evaluation (severity of the injury and effects of the therapy) of ischemic damage. If so, this may lead to the hypothesis of renalase being the ubiquitous anti-ischemic agent, regardless of the tissue. Moreover, it was further suggested that renalase promoted cell protection by activation of sirtuin 1 (SIRT1) and that renalase administration significantly alleviates liver ischemic injury. This seems feasible, knowing that SIRT1 activation requires nicotinamide adenine dinucleotide (NAD+) and that renalase was proven to oxidize α-NADP, converting it to β-NAD^+^ (33). Nevertheless, the deprivation of the cellular NAD/NADH ratio may lead to significant myocardial ischemic injury, as observed in experimental models of renalase deficiency (18). Moreover, SIRT-1 is documented to exert protection against cardiac ischemic damage (34), and it may be presumed that is, at least partially, achieved by renalase action.

Increasing evidence implicates that renalase cytokine traits are crucial for its protective role; however, in light of the pleiotropic role of renalase, its properties in catecholamine metabolism should also be discussed. In several recent studies, it was confirmed that nicotine, dopamine, and epinephrine may initiate substantial renalase gene expression in different tissues (3, 27, 30), whereas a catecholamine surge from the ischemic tissue triggers renalase secretion (14). The sympathetic nervous system has been heavily involved in the pathogenesis of chronic HF, resulting in low LVEF; accordingly, renalase plasma levels are likely compensatorily increased to counteract the chronic stimulation of adrenergic receptors. Moreover, increased catecholamine levels have been significantly associated with cardiac ischemia, whether acute or chronic (13). It is known that activation of both α-adrenergic receptors results in significant organ damage; therefore, their “renalase-mediated blockage” warrants anti-ischemic protection, as verified in the animal model (17) and also allegedly in humans. In the same manner, renalase is suggested to act as a β-adrenergic receptor “blocker” (3), providing decreased blood pressure, cardiac contractility, and heart rate (3). All things considered, both catecholamines and NAD^+^ may presumably be involved in renalase anti-ischemic properties, although the exact underlying pathway is not fully defined yet (25, 26).

As our results document, we also tested and validated the power of renalase for the prediction of exercise-induced ischemia in the total cohort of chronic HF and with specific regard to LVEF rate. The discriminatory potential of renalase for ischemia prediction proved non-inferiority compared to that of BNP and was similar to those of cardiac remodeling biomarkers in the total HF cohort. Moreover, among total chronic HF patients, those with reduced LVEF presenting with higher renalase levels were more likely to develop ischemic ECG changes during the exercise stress test, even though they were all without overt chest pain, compared to the HFpEF phenotype. In addition to these findings, renalase gene polymorphism (Glu37Asp) was associated with poor exercise capacity and significant exercise-inducible ischemia in stable CAD patients (13). Indeed, renalase knockout animals badly tolerated induced ischemic insult with the subsequent cardiac lesion. This happens presumably because of renalase response feasibility to impede catecholamine surge accumulation in the myocardial tissue and to accelerate its removal (18) and promptly provide antiapoptosis, anti-inflammation, and antioxidation, through the tumor necrosis factor α/NF-κβ pathway (35). As evidenced, renalase is up-regulated under pathologic stimuli, chronic hypoxia, and acute ischemia, to promote cardiomyocytes survival (35); therefore, treatment with renalase therapy is worthy of research.

Taken together, the authors may not evidence the question as to whether renalase multifold elevation in chronic HF patients, predominantly in the HFrEF phenotype, represents a compensatory phenomenon against hypoxia/ischemia and whether it employs beneficial effects for the patients (or it is a pathological event in itself). We may, however, assume that this rise is not transient but permanent, most likely in an effort to “overcome” hypoxia. Another task to be clarified might be the determination of the reference values for renalase elevation in CHF, cutoffs for differentiation between HF phenotypes, and identification of possible triggers (if any) for renalase decline. Additionally, determination of the cutoff points for differentiation between chronic (stable CHF) and acute ischemia may prove its clinical validity. It would also be intriguing to establish the possible association of renalase and exercise-induced B-lines during exercise stress echocardiography, knowing that B-lines are easy to measure, frequent, and commonly increase during exercise stress echocardiography, providing a piece of significant information about functional impairment (at rest and during stress) in the short-term follow-up (20, 36).

To the extent of our knowledge, these findings represent some originality regarding the discriminative potential and positive prognostic ability of renalase for the prediction of ischemia in HF patients. Brain natriuretic peptide alone has limited specificity for heart functional abnormalities detection (37, 38); therefore, an integrative approach using more biomarkers warrants better identification of the patients at risk for a bad outcome, with renalase possibly being among them. For instance, the most recent study (37) evidenced that BNP did not increase discrimination for diastolic dysfunction in the HF cohort, whereas among the four biomarkers evaluated (BNP, Gal-3, sST2, and N-terminal propeptide of procollagen type III), galectin-3 demonstrated better discriminatory potential compared to that in BNP.

Accordingly, assumed as a peripheral blood biomarker for ischemia, it may add the diagnostic validity to the standard testing, enabling timely identification of patients without chest pain who are likely to develop ischemia or the recognition of patients presenting with silent ischemia. Knowing that discriminatory ability of renalase for ischemia prediction in patients with HF, regardless of the ejection fraction, was similar to those of BNP, sST2, galectin-3, and GDF-15, we are not offering renalase as a sole marker of ischemia prediction, but implying its potential contribution to ischemia-risk stratification, through multiple biomarker protocols.

### Study Limitations

The present study has several limitations. The most important limitation of the study was certainly the relatively small number of patients included, mostly due to strict exclusion criteria. We, however, wanted to provide a clinical group whose biomarker plasma levels were essentially related to HF. Therefore, the exclusion of almost 50 participants, owing to their comorbidities (kidney failure, liver cirrhosis, malignant disease, etc.), left us with a relatively small number of eligible participants, which possibly resulted in reduced statistical significance. Second, the determination of HF to that of reduced (HFrEF) and preserved LVEF (HFpEF) was obtained out of the 2016 ESC guidelines differentiation of HF into the three subgroups of patients (1). If we had chosen to further divide our study sample into the three subgroups, it would have resulted in even more reduced statistical significance. However, the ACC/AHA guidelines, which were extensively updated in 2013 (2) and had focused updates in 2016 and 2017, still define HF as HFrEF and HFpEF; therefore, our clinical group was categorized in the same manner. Moreover, serial renalase measurements (at least before and after an ischemic episode) certainly add substantial statistical and clinical value for biomarkers in order to be prognostic and might improve the results of the study, but according to the study design were not performed. Henceforth, catecholamine determination could support further clarification of their possible interrelation with renalase, as well as renalase correlations with routinely performed methods for ischemia assessment. Finally, the cross-sectional design of the study did not allow conclusions as to whether renalase may be a predictor for future adverse events or improved outcomes, so prospective studies should confirm and validate these findings. For these reasons, this study should be observed as a well-considered hypothesis-generating subsequent large-scale research.

## Conclusion

In summary, our research is the first of a sort to assess plasma renalase in CHF patients with regard to ejection fraction stratification. Increased plasma renalase demonstrates to be an independent predictor of ischemia induced by exercise stress testing on top of evaluated cardiac remodeling biomarkers (sST2, galectin-3, GDF-15, and syndecan-1) and cystatin C, but does not reach plasma BNP, in both analyzed groups, the cohort of the total HF and HFrEF phenotype.

However, the comparative analysis of their discriminatory values for ischemia prediction evidences that in the total HF group, BNP plasma concentration does not demonstrate significantly better discrimination compared to that of renalase, galectin-3, and GDF-15. In the HFrEF subtype, plasma BNP proved significantly better discriminatory potential compared to all evaluated biomarkers, including renalase, whereas the discriminatory ability of renalase was significantly better compared to those of galectin-3 and GDF-15 and similar to those of sST2 and syndecan-1. The obtained results clearly indicate that plasma renalase emerges to be a non-inferior biomarker in the prediction of ischemia in the HF cohort, compared to plasma BNP, emphasizing the relevance for the establishment of their subsequent comparative prognostic analyses and further confirmatory studies.

Renalase seems to be a feasible addition to the multiple biomarker strategy for the improvement of conventional markers' predictive potential or possibly differentiating phenotypes in CHF or ischemia prediction in patients with HF. For these reasons, renalase should be investigated much more comprehensively.

## Data Availability Statement

The raw data supporting the conclusions of this article will be made available by the authors, without undue reservation.

## Ethics Statement

The studies involving human participants were reviewed and approved by the Faculty of Medicine, Nis, University Nis (12-10580-2/3) and the Institute for Treatment and Rehabilitation Niska Banja, Niska Banja (03-4185/1). The patients/participants provided their written informed consent to participate in this study.

## Author Contributions

DS designed the protocol of the study and with all the authors participated in the collection, interpretation, analysis of the data, searching the literature, drafting of the manuscript, critical review of the article, and approved the final version for publication.

## Conflict of Interest

The authors declare that the research was conducted in the absence of any commercial or financial relationships that could be construed as a potential conflict of interest.

## Publisher's Note

All claims expressed in this article are solely those of the authors and do not necessarily represent those of their affiliated organizations, or those of the publisher, the editors and the reviewers. Any product that may be evaluated in this article, or claim that may be made by its manufacturer, is not guaranteed or endorsed by the publisher.
